# Cognitive Function is a Predictor of the Daily Step Count in Patients With Subacute Stroke With Independent Walking Ability: A Prospective Cohort Study

**DOI:** 10.1016/j.arrct.2021.100132

**Published:** 2021-05-15

**Authors:** Daisuke Ito, Michiyuki Kawakami, Yuya Narita, Taiki Yoshida, Naoki Mori, Kunitsugu Kondo

**Affiliations:** a Department of Rehabilitation Medicine, Tokyo Bay Rehabilitation Hospital, Chiba, Japan; b Department of Rehabilitation Medicine, Keio University School of Medicine, Tokyo, Japan; c Graduate School of Human Sciences, Waseda University, Tokorozawa City, Saitama, Japan

**Keywords:** Cognition, Exercise, Rehabilitation, Stroke, Walking, *List of abbreviations:* CI, confidence interval, MCI, mild cognitive impairment, MMSE, Mini-Mental State Examination

## Abstract

**Objectives:**

To investigate the physical, cognitive, and psychological factors related to daily step count in patients with subacute stroke.

**Design:**

Prospective cohort study.

**Setting:**

A subacute rehabilitation ward with 160 beds.

**Participants:**

Patients with subacute stroke who could walk independently (N=101). Among the 101 participants enrolled in this study (mean age, 64.5±13.5y), 64.4% (n=65) were men and 69.3% (n=70) were patients with cerebral infarction.

**Interventions:**

We assessed ambulatory activity using a pedometer placed in the pants pocket on the nonparalyzed side continuously for 7 consecutive days. We also obtained demographic and clinical information and recorded the following measurement scores: Stroke Impairment Assessment Set, FIM, Mini-Mental State Examination (MMSE), Self-Rating Depression Scale, and Apathy Scale. All measurements were collected at admission and discharge.

**Main Outcome Measures:**

The outcomes assessed were ambulatory activity, motor and sensory functions, functional disability, cognitive function, depressive symptoms, and motivation.

**Results:**

The median daily steps ambulated at admission and discharge were 5584 steps (interquartile range, 3763-7096 steps) and 5991 steps (interquartile range, 4329-8204 steps), respectively. In the univariate regression analysis, age, sex, serum albumin level, affected side of the brain, and MMSE score at admission were significantly associated with the daily step count at discharge. Multiple regression analysis using these 5 items as independent variables revealed that the MMSE score at admission (reference, 28-30 points; B, −2.07; 95% confidence interval, −3.89 to −0.35; β, −0.22; *P*=.027) was significantly associated with the daily step count at discharge.

**Conclusions:**

Cognitive function at admission had a significant association with the daily step count at discharge in patients with subacute stroke who could walk independently.

## Highlights

• Cognition at admission may predict daily step count.
• Cognitive impairment may increase risk of poor ambulation after subacute stroke.
• Ambulation poststroke is influenced by both physical and cognitive factors.

Stroke is a leading cause of disability,^1^^,^^2^ and poststroke disability improves after rehabilitation.^3^^,^^4^ Exercise-based therapy is widely known to enhance motor recovery after stroke, with evidence of the positive relationship between physical activity and recovery in a meta-analysis.^5^ Moreover, physical activity in patients with stroke has been reported to be associated with the prevention of stroke recurrence,^6^^,^^7^ prevention of readmission,^8^ and improvement of physical health 1-year poststroke.^9^ Thus, it is recommended to perform physical activity during the rehabilitation period.^10^

Although patients with stroke in the subacute phase undergo intensive rehabilitation to improve activities of daily living, with the expectation that it will help speed up recovery,^11^ a systematic review reported that patients with subacute stroke are often inactive.^12^ The number of steps taken by patients with subacute stroke is fewer than those taken by healthy adults^12^^,^^13^ and patients with chronic stroke.^14^ Furthermore, daily steps during hospitalization are related to those after discharge.^15^^,^^16^ Therefore, established exercise habits are important, especially in the subacute rehabilitation hospital.

Very few reports have investigated the various factors associated with the daily step count in patients with subacute stroke. To our knowledge, only physical aspects such as walking speed^17^ and physical function^18^ are reportedly associated with the daily steps taken by patients with subacute stroke. We hypothesized that cognitive and psychological aspects, such as mild cognitive decline and motivation decline, also affect daily steps. Exploring various factors related to daily steps, including physical, cognitive, and psychological aspects, is important for establishing exercise habits in the subacute setting. In particular, patients with mild stroke who can walk independently usually have less support after home discharge; thus, it is necessary to investigate factors related to inactivity. However, the relationship between daily steps and physical, cognitive, and psychological aspects remains unclear in patients with subacute stroke with independent walking ability. Therefore, this study aimed to explore the factors associated with the daily step count in patients with subacute stroke who could walk independently, including physical, cognitive, and psychological factors.

## Methods

### Study design and participants

This prospective cohort study was conducted in adherence to the Strengthening the Reporting of Observational Studies in Epidemiology statement. This study included patients who were admitted to subacute rehabilitation wards between January 1, 2018 and July 31, 2019. The inclusion criteria were aged ≥20 years and admission with a first-ever stroke. The exclusion criteria included subarachnoid hemorrhage, cognitive impairment (Mini-Mental State Examination [MMSE] score ≤23 points), aphasia, early discharge (within 7 days of admission), hospital transfer, refusal to participate, and nonindependent walking during hospitalization. This study was conducted in accordance with the Declaration of Helsinki, and this study was reviewed and approved by the Ethics Committee of Tokyo Bay Rehabilitation Hospital (#189). All participants provided written informed consent before data collection.

### Data collection

The following demographic data were collected from the medical records: age (y), sex (men or women), body mass index (kg/m^2^), serum albumin level (g/dL), type of stroke (cerebral infarction or cerebral hemorrhage), side of the brain affected (right or left), duration from the stroke onset to admission (d), duration of hospitalization (d), circumstances of living (alone or not), discharge disposition (home or facility), and presence of neglect (yes or no). Data regarding duration of hospitalization and condition at discharge were collected at discharge, whereas the other data were collected at admission. The measurement scores recorded at admission and discharge are presented below. Assessments were completed within 1 week of admission and discharge.

### Ambulatory activity

Ambulatory activity was operationally defined as the daily step count measured using a pedometer. In previous studies, ambulatory activity was assessed using a pedometer in patients with stroke, and this measurement method had an established feasibility,^19^, ^20^, ^21^ validity,^22^^,^^23^ and reliability.^23^ We used a pedometer^a^ with a 14-day data storage capacity that had a triaxial acceleration sensor that could measure daily steps. The pedometer was placed in the pants pocket on the nonparalyzed side. This pedometer has not been validated in patients with stroke; however, a previous study reported on the validity of the same measurement method as this study.^22^ Patients were instructed to wear the pedometer during the day continuously for 7 consecutive days except while bathing and sleeping. The average daily steps tracked for at least 3 days was used for each patient in this study.^24^ The primary outcome measure was the average daily steps at discharge.

### Walking speed

Walking speed was assessed using a 10-m walking test, which has established reliability in patients with stroke.^25^ Participants were instructed to walk at a comfortable speed, and the time required to walk from the starting line to the goal line was measured using a stopwatch. Walking speed was calculated by dividing the 10-m distance by the time required (m/s).

### Stroke impairment assessment set

Motor and sensory functions were assessed using the stroke impairment assessment set.^26^ Motor scores consist of 2 tests for upper extremity (0-10) and 3 tests for the lower extremity (0-15).^27^ Sensory scores evaluate superficial sensation and deep sensation of the affected upper (0-6) and lower (0-6) extremities.^27^ Higher scores represent better functions.

### FIM

Functional disability was assessed using the FIM.^28^ The FIM comprises 13 motor subscales (FIM motor) and 5 cognitive subscales (FIM cognitive). The FIM motor consists of the following 4 categories: self-care (eating, grooming, bathing, dressing-upper body, dressing-lower body, toileting), sphincter control (bladder management and bowel management), transfers (bed/chair/wheelchair, toilet, tub/shower), and locomotion (walk/wheelchair and stairs). The FIM cognitive consists of the following 2 categories: communication (comprehension and expression) and social cognition (social interaction, problem solving, memory). Each item has a 7-grade scale ranging from 1 point (total assistance or not testable) to 7 points (complete independence). The total possible score is 18-126 points, 13-91 points, and 5-35 points for the total FIM, FIM motor, and FIM cognitive, respectively, with a higher score representing greater functional independence. The severity of function disability at discharge was categorized as mild (FIM motor≥62 points), moderate (FIM motor=38-61 points), or severe (FIM motor≤37 points) based on a previous study.^29^

### Mini-Mental State Examination

Cognitive function was assessed using the MMSE.^30^ It consists of 11 items as follows (maximum score of each item): orientation to time (5), orientation to place (5), registration of 3 words (3), attention and calculation (serial sevens or spelling) (5), recall (3), naming (2), repetition (1), comprehension of verbal (3), comprehension of written (1), writing (1), and construction (1). The maximum possible score is 30 points, and scores ≤23 points represent moderate to severe cognitive problems,^30^ 24-27 points represent mild cognitive impairment (MCI),^31^, ^32^, ^33^ and 28-30 points represent normal cognitive function.

### Self-Rating Depression Scale

Depressive symptoms were assessed using the Self-Rating Depression Scale, consisting of 20 items.^34^ Each question is scored on the following 4-point scale: 1 point, rarely; 2 points, sometimes; 3 points, commonly; and 4 points, most of the time. Total possible scores are 20-80 points, with a higher score indicating more depressive symptoms; the cutoff value is 50 points.^35^ The validity of the Self-Rating Depression Scale has been established in patients with stroke.^36^

### Apathy Scale

Motivation was assessed using the Apathy Scale,^37^ which consists of 14 items. Each item is scored on the following 4-point scale: 0 points, not at all; 1 point, slightly; 2 points, some; and 3 points, commonly. Total possible scores are 0-42 points, with a higher score indicating more apathy symptoms; the cutoff value is 16 points.^37^ The Apathy Scale has established validity and reliability in patients with stroke.^38^

### Statistical analysis

The normality of the data was plotted using histograms and assessed using the Shapiro-Wilk test. To assess the factors associated with the daily step count at discharge, we used multiple regression analysis to determine the partial regression coefficients (B), 95% confidence interval [CI], standard partial regression coefficients (β), and variance inflation rate. Daily step count at discharge was the dependent variable. Daily steps at discharge were divided by 1000 to prevent the partial regression coefficient from becoming too large.^6^ Independent variables were the factors with a *P*≤.05 in the univariate regression analysis. Independent variables without normality were categorized by cutoff values. All statistical analyses were performed using SPSS Statistics 21.0.^b^ Values of *P*≤.05 were considered statistically significant.

## Results

**Fig 1:**
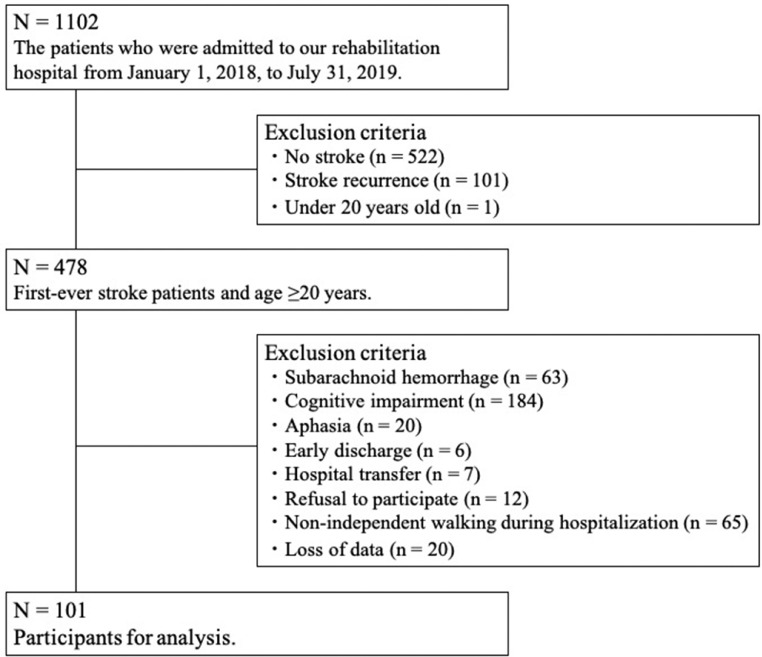
Flowchart of the patient selection process.

Of the 478 patients with stroke screened for eligibility, 377 were excluded because of subarachnoid hemorrhage (n = 63), cognitive impairment (n = 184), aphasia (n = 20), early discharge (n = 6), hospital transfer (n = 7), refusal to participate (n = 12), nonindependent walking during hospitalization (n = 65), or loss of data (n = 20). Thus, a total of 101 participants were enrolled (fig 1).

The characteristics of the study participants are shown in table 1. The mean age was 64.5±13.5 years. The proportions of men and patients with cerebral infarction were 64.4% (n=65) and 69.3% (n=70), respectively. The measurement scores of the study participants are shown in table 2. The median daily steps at discharge, FIM motor score at admission, and MMSE score at admission, were 5991 steps (interquartile range, 4329-8204 steps), 63 points (interquartile range, 51-75 points), and 28 points (interquartile range, 27-29 points), respectively.

**Table 1:** Characteristics of the study participants

Characteristics	Overall N=101
Age (y), mean ± SD	64.5±13.5
Sex (men), n (%)	65 (64.4)
BMI, mean ± SD	23.2±3.9
Serum albumin level (g/dL), mean ± SD	3.9±0.4
Stroke type (cerebral infarction), n (%)	70 (69.3)
Brain side affected (left), n (%)	48 (47.5)
Duration from stroke onset to admission (d), median (IQR)	26 (20-35)
Hospital duration (d), median (IQR)	58 (37-100)
Living situation (alone), n (%)	10 (9.9)
Discharge disposition (home), n (%)	99 (98.0)
Neglect (yes), n (%)	7 (6.9)

Abbreviations: BMI, body mass index (calculated as weight in kilograms divided by height in meters squared); IQR, interquartile range.

**Table 2:** Scores of the study participants in the measures used

Measures	Admission	Discharge
Daily steps, median (IQR)	5584 (3763-7096)	5991 (4329-8204)
Walking speed (m/s), mean ± SD	0.9±0.4	1.1±0.4
SIAS U/E motor score (0-10), median (IQR)	8 (5-10)	9 (8-10)
SIAS L/E motor score (0-15), median (IQR)	13 (10-15)	14 (12-15)
SIAS U/E sensory score (0-6), median (IQR)	6 (4-6)	6 (6-6)
SIAS L/E sensory score (0-6), median (IQR)	6 (4-6)	6 (5-6)
FIM motor score, median (IQR)	63 (51-75)	88 (83-90)
FIM cognitive score, median (IQR)	30 (26-32)	34 (31-35)
FIM total score, median (IQR)	92 (80-103)	121 (116-124)
MMSE score, median (IQR)	28 (27-29)	29 (28-30)
SDS score, median (IQR)	39 (32-45)	36 (31-44)
Apathy Scale score, median (IQR)	10 (7-15)	11 (6-15)

Abbreviations: IQR, interquartile range; L/E, lower extremity; SDS, Self-Rating Depression Scale; SIAS, stroke impairment assessment set; U/E, upper extremity.

Table 3 shows the results of the univariate and multiple regression analysis. In univariate regression analysis, age (B, −0.10; 95% CI, −0.16 to −0.03; β, −0.29; *P*=.003), sex (reference, women; B, 3.09; 95% CI, 1.35-4.83; β, 0.33; *P*=.001), serum albumin level at admission (B, 3.57; 95% CI, 1.18-5.95; β, 0.29; *P*=.004), affected side of the brain (reference, left; B, 1.95; 95% CI, 0.22-3.68; β, 0.22; *P*=.027), and MMSE score at admission (reference, 28-30 points; B, −3.29; 95% CI, −5.05 to −1.54); β, −0.35; *P<*.001) were significantly associated with the daily step count at discharge. Furthermore, multiple regression analyses were performed to identify the variables associated with mean daily steps at discharge. The independent variables at admission were age, sex, serum albumin level at admission, side of the brain affected, and MMSE score at admission, which were considered significant by univariate regression analysis. The factor associated with the daily steps at discharge was MMSE score at admission (reference, 28-30 points; B, −2.07; 95% CI, −3.89 to −0.35; β, −0.22; *P*=.027). There were no other factors with variance inflation rate <10.

**Table 3:** Univariate and multiple regression analysis of the daily step count

Variable at admission		Univariate Regression					Multiple Regression					
		B	95% CI		β	*P* Value	*B*	95% CI		*β*	*P* Value	VIF
			Lower	Upper				Lower	Upper			
Age		−0.10	−0.16	−0.03	−0.29	.003*	−0.03	−0.10	0.04	−0.08	.442	1.46
Sex	Men	3.09	1.35	4.83	0.33	.001*	1.59	−0.22	3.39	0.17	.084	1.21
	Women		1.00					1.00				
BMI		0.11	−0.12	0.34	0.10	.333						
Serum albumin level		3.57	1.18	5.95	0.29	.004*	2.20	−0.39	4.79	0.18	.095	1.38
Stroke type	Hemorrhage	0.44	−1.47	2.36	0.05	.648						
	Infarction		1.00									
Brain side affected	Right	1.95	0.22	3.68	0.22	.027†	1.39	−0.27	3.05	0.16	.099	1.11
	Left		1.00					1.00				
Duration from stroke onset to admission		−0.02	−0.07	0.04	−0.06	.563						
Living situation	Alone	0.20	−0.58	0.99	0.05	.612						
	Not alone		1.00									
Neglect	Yes	−2.46	−5.91	0.99	−0.14	.160						
	No		1.00									
Walking speed		2.40	−0.05	4.85	0.19	.055						
SIAS U/E motor score	0-7	−1.48	−3.29	0.34	−0.16	.110						
	8-10		1.00									
SIAS L/E motor score	0-12	−0.33	−2.27	1.61	−0.03	.736						
	13-15		1.00									
SIAS U/E sensory score	0-5	1.14	−0.66	2.94	0.13	.210						
	6		1.00									
SIAS L/E sensory score	0-5	0.62	−1.17	2.41	0.07	.494						
	6		1.00									
FIM motor score	13-37	−3.32	−7.00	0.37	−0.18	.077						
	38-61	−1.76	−3.53	0.01	−0.19	.051						
	62-91		1.00									
FIM cognitive score	<23	−0.20	−3.68	3.29	−0.01	.911						
	≥23		1.00									
MMSE score	24-27	−3.29	−5.05	−1.54	−0.35	<.001*	−2.07	−3.89	−0.35	−0.22	.027†	1.20
	28-30		1.00									
SDS score	≥50	0.62	−2.12	3.35	0.05	.655						
	<50		1.00									
Apathy Scale score	≥16	0.48	−1.60	2.55	0.05	.649						
	<16		1.00									
Adjusted *R*^2^							0.20					

NOTE. Model F test: *P*<.001. Dependence variable: daily steps at discharge (per 1000 steps).

Abbreviations: BMI, body mass index (calculated as weight in kilograms divided by height in meters squared); L/E, lower extremity; SDS, Self-Rating Depression Scale; SIAS, stroke impairment assessment set; U/E, upper extremity; VIF, variance inflation rate.

⁎ *P*<.010.

† *P*<.050.

## Discussion

We investigated the factors associated with the daily step count at discharge in patients with subacute stroke with independent walking ability. In the multiple regression analysis, we demonstrated that the MMSE score at admission was mildly but significantly associated with the daily step ambulated at discharge.

We found that the MMSE score at admission was related with the daily steps in multiple regression analysis. To our knowledge, there have been no studies that have investigated the relationship between cognitive function and ambulatory activity in patients with subacute stroke. For patients with chronic stroke, however, cognitive impairment is a risk factor for inactivity.^39^ In the present study, we excluded patients with MMSE scores ≤23 points because patients with severe cognitive impairment were omitted. Our results suggested that even MCI affected ambulatory activity in patients with subacute stroke. According to the available data, this is the first study to demonstrate that the MCI at admission was associated with the daily step count at discharge in patients with subacute stroke. It has been reported that MCI was associated with low physical activity in older adults.^40^, ^41^, ^42^ Similarly, in patients with stroke, MCI may interfere with active participation in rehabilitation and voluntary practices. Thus, cognitive screening tests to identify MCI and approaches to increase their activity may be important.

One strength of this study is that we identified factors associated with the daily step count in patients with subacute stroke in the multiple regression analysis, including cognitive aspects such as cognitive function, motivation, and depressive symptoms. Previous studies have reported that daily step count was related to only physical aspects^17^^,^^18^ and did not clarify the influence of confounding factors on daily step count. Thus, our results after the adjustment for physical and cognitive confounding factors were novel.

### Study limitations

There are some limitations to this study. First, the study was conducted at a single facility, which limits the generalizability of the results. Second, we used the psychological scale; thus, we excluded patients with a MMSE score ≤23 points. Finally, we could not measure activity time, content, or intensity because we used only a pedometer to measure daily steps. Nevertheless, the pedometer was easy to operate; hence, many patients with stroke agreed to participate in this study. Furthermore, the number of daily steps could be confirmed and used as a motivation for self-practice. In the future, longitudinal studies are needed to determine whether the ambulatory activity is maintained after discharge.

## Conclusions

We found that cognitive function at admission was significantly associated with the daily step count at discharge in patients with subacute stroke who could walk independently. Therefore, patients with stroke with MCI may be at risk for poor ambulatory activity.

## Suppliers

a Pedometer, YAMASA EX-300; Yamasa Tokei Keiki Co, Ltd.
b SPSS Statistics 21.0; IBM.

## Appendix Supplementary materials

Image, application 1
