# Supplementary material for: Cognitive Function is a Predictor of the Daily Step Count in Patients With Subacute Stroke With Independent Walking Ability: A Prospective Cohort Study
Source: Arch Rehabil Res Clin Transl. 2021 May 15;3(3):100132. doi: 10.1016/j.arrct.2021.100132 (PMC8463495; doi:10.1016/j.arrct.2021.100132)
Supplement: Supplementary file 1 [file mmc1.pdf]

editage

Editing Certificate

This document certifies that the manuscript listed below has been edited to ensure language and grammar accuracy and is error free in these aspects. The logical presentation of ideas and the structure of the paper were also checked during the editing process. The edit was performed by professional editors at Editage, a division of Cactus Communications. The author's core research ideas were not altered in any way during the editing process. The quality of the edit has been guaranteed, with the assumption that our suggested changes have been accepted and the text has not been further altered without the knowledge of our editors.

MANUSCRIPT TITLE

Cognitive function is a predictor of the daily step count in subacute stroke patients with independent walking ability: A prospective cohort study

AUTHORS

Daisuke Ito

ISSUED ON

April 26, 2021

JOB CODE

CJMYJ_2_8

THE EDITAGE Promise QUALITY GUARANTEE

Vikas Narang

Vikas Narang
Chief Operating Officer - Editage

editage

CACTUS

Editage, a brand of Cactus Communications, offers professional English language editing and publication support services to authors engaged in over 1300 areas of research. Through its community of experienced editors, which includes doctors, engineers, published scientists, and researchers with peer review experience, Editage has successfully helped authors get published in internationally reputed journals. Authors who work with Editage are guaranteed excellent language quality and timely delivery.

IMPACT SCIENCE impact.science

researcher.life

CACTUS LIFE SCIENCES lifesciences.cactusglobal.com

GLOBAL :
(833) 979-0061 | request@editage.com

JAPAN :
0120-50-2987 | submissions@editage.com
